# Feasibility Study of a Digital Screen-Based Calming Device for Managing BPSD During Care

**DOI:** 10.1093/geroni/igaa057.2722

**Published:** 2020-12-16

**Authors:** Gloria Gutman, Avantika Vashisht, Taranjot Kaur, Ryan Churchill, Amir Moztarzadeh, Mojgan Karbakhsh

**Affiliations:** 1 Simon Fraser University, Vancouver, British Columbia, Canada; 2 Simon Fraser University Gerontology Research Centre, Vancouver, British Columbia, Canada

## Abstract

MindfulGarden (MG) is a digital device resembling a flat screen TV, with touchless sensors that react to voice and motion. In this study 13 long-term care home residents aged 74-100 exhibiting Behavioural and Psychological Symptoms of Dementia (BPSD) were randomized to treatment and control groups. On days 1-3 the treatment group received usual care plus exposure to MG during morning and evening care - events well documented to be problematic for residents and care staff; controls received usual care only. On day 4 both groups were exposed to MG with verbal cueing. A 26-item checklist was used to record frequency and types of disruptive BPSD exhibited; care duration was recorded in minutes. There was a trend toward reduction of BPSD and duration of care during morning care. Findings suggest that verbal cueing may be important for successful implementation of MG in calming residents with dementia during routine care.

